# Estrogen Receptor Beta rs1271572 Polymorphism and Invasive Ovarian Carcinoma Risk: Pooled Analysis within the Ovarian Cancer Association Consortium

**DOI:** 10.1371/journal.pone.0020703

**Published:** 2011-06-06

**Authors:** Galina Lurie, Lynne R. Wilkens, Pamela J. Thompson, Yurii B. Shvetsov, Rayna K. Matsuno, Michael E. Carney, Rachel T. Palmieri, Anna H. Wu, Malcolm C. Pike, Celeste L. Pearce, Usha Menon, Aleksandra Gentry-Maharaj, Simon A. Gayther, Susan J. Ramus, Alice S. Whittemore, Valerie McGuire, Weiva Sieh, Paul D. P. Pharoah, Honglin Song, Jacek Gronwald, Anna Jakubowska, Cezary Cybulski, Jan Lubinski, Joellen M. Schildkraut, Andrew Berchuck, Susanne Krüger Kjær, Estrid Høgdall, Peter A. Fasching, Matthias W. Beckmann, Arif B. Ekici, Alexander Hein, Georgia Chenevix-Trench, Penelope M. Webb, Jonathan Beesley, The Australian Ovarian Cancer Study and Australian Cancer Study Groups, Marc T. Goodman, The Ovarian Cancer Association Consortium

**Affiliations:** 1 Cancer Epidemiology Program, University of Hawaii Cancer Center, Honolulu, Hawaii, United States of America; 2 Department of Epidemiology, Johns Hopkins Bloomberg School of Public Health, Baltimore, Maryland, United States of America; 3 Department of Obstetrics and Gynecology, John A. Burns School of Medicine, University of Hawaii, Honolulu, Hawaii, United States of America; 4 Department of Community and Family Medicine, Duke University Medical Center, Durham, North Carolina, United States of America; 5 Department of Preventive Medicine, Keck School of Medicine, University of Southern California Norris Comprehensive Cancer Center, Los Angeles, California, United States of America; 6 Department of Epidemiology and Biostatistics, Memorial Sloan-Kettering Cancer Center, New York, New York, United States of America; 7 Department of Gynecological Oncology, University College London EGA Institute for Women's Health, London, United Kingdom; 8 Department of Health Research and Policy, Stanford University School of Medicine, Stanford, California, United States of America; 9 Department of Oncology, University of Cambridge, Cambridge, United Kingdom; 10 Department of Genetics and Pathology, International Hereditary Cancer Center, Pomeranian Medical University, Szczecin, Poland; 11 Department of Viruses, Hormones and Cancer, Danish Cancer Society, Institute of Cancer Epidemiology, Copenhagen, Denmark; 12 Gynecologic Clinic, The Juliane Marie Centre, Rigshospitalet, University of Copenhagen, Copenhagen, Denmark; 13 Division of Hematology and Oncology, David Geffen School of Medicine, University of California at Los Angeles, Los Angeles, California, United States of America; 14 Department of Gynecology and Obstetrics, University Hospital Erlangen, Friedrich-Alexander University Erlangen-Nürnberg, Nürnberg, Germany; 15 Institute of Human Genetics, Friedrich-Alexander University Erlangen-Nürnberg, Nürnberg, Germany; 16 Division of Genetics and Population Health, Queensland Institute of Medical Research, Brisbane, Australia; 17 Queensland Institute of Medical Research, Brisbane, Australia and Peter MacCallum Cancer Study, Melbourne, Australia; Cornell University, United States of America

## Abstract

The association of ovarian carcinoma risk with the polymorphism rs1271572 in the *estrogen receptor beta* (*ESR2*) gene was examined in 4946 women with primary invasive ovarian carcinoma and 6582 controls in a pooled analysis of ten case-control studies within the Ovarian Cancer Association Consortium (OCAC). All participants were non-Hispanic white women. Odds ratios (ORs) and 95% confidence intervals (CIs) were estimated using unconditional logistic regression adjusted for site and age. Women with the *TT* genotype were at increased risk of ovarian carcinoma compared to carriers of the *G* allele (OR = 1.10; 95%; CI: 1.01–1.21; p = 0.04); the OR was 1.09 (CI: 0.99–1.20; p = 0.07) after excluding data from the center (Hawaii) that nominated this SNP for OCAC genotyping A stronger association of rs1271572 *TT* versus *GT/GG* with risk was observed among women aged ≤50 years versus older women (OR = 1.35; CI: 1.12–1.62; p = 0.002; p for interaction = 0.02) that remained statistically significant after excluding Hawaii data (OR = 1.34; CI: 1.11–1.61; p = 0.009). No heterogeneity of the association was observed by study, menopausal status, gravidity, parity, use of contraceptive or menopausal hormones, tumor histological type, or stage at diagnosis. This pooled analysis suggests that rs1271572 might influence the risk of ovarian cancer, in particular among younger women.

## Introduction

The mitogenic action of estrogen appears critical to the etiology and progression of human gynecologic cancers [1]. The principal biological activities of estrogens are to influence the growth, differentiation, and function of reproductive tissues. Estrogens interact with their receptors to mediate various signaling pathways that are likely associated with the risk of ovarian cancer [2]. Estrogen receptors exist in two forms, estrogen receptor alpha (ERα) and estrogen receptor beta (ERβ) [3] which is the predominant estrogen receptor in the ovary [4]–[6]. Although the exact role of ERβ in ovarian carcinogenesis remains to be determined, recent *in vivo* and *in vitro* studies suggest that ERβ is involved with the control of cellular proliferation, motility and apoptosis in ovarian cancer; and loss of ERβ expression is associated with tumor progression [7]–[10].

The human ERβ gene (*ESR2*) is located on chromosome 14q23.2 spanning ∼61.2 kb. Previously, the multiethnic Hawaiian Ovarian Cancer Study evaluated four single nucleotide polymorphisms (SNPs) in the *ESR2* gene, as well as their associated haplotypes, in relation to risk of borderline and invasive ovarian cancer and found that rs1271572 in the promoter region of the gene may be an ovarian carcinoma susceptibility marker [11]. The homozygous variant genotype (*TT*) carriers had a 79% increase in risk (95% CI: 1.15–2.79) compared to women with the *GG* genotype. White women had more than a two-fold increase in risk (OR = 2.42; 95% CI: 1.14–5.15). In the present study, we performed a replication analysis of our putative significant findings by genotyping rs1271572 among participants in nine additional studies within the Ovarian Cancer Association Consortium (OCAC), a forum for researchers to evaluate promising genetic associations with ovarian cancer with increased power [12], [13]. To minimize the effects of population stratification, this study included only white non-Hispanic women from developed countries with comparable ovarian cancer incidence rates. Only cases with invasive tumors were included.

## Results

The mean age of cases (57.3 years; standard error = 0.2) and controls (57.2 years; standard error = 0.1) was similar (Table 1). Minor allele frequencies among controls ranged from 0.41 to 0.46 with no statistically significant differences in genotype distribution among studies (p = 0.69) (Table S1) overall and by age group.

**Table 1:** Description of the studies included in the pooled analysis of the *ESR2* rs1271572 and ovarian carcinoma risk.

Study Name	Location	Study Design	White non-Hispanic women			
			Cases (invasive)		Controls	
			N	Mean age (SE), yrs	N	Mean age (SE), yrs
AUS (Australian National Ovarian Cancer Study)	Australia	Population-based case-control	1051	58.5 (0.3)	1148	56.7 (0.3)
BAV (Bavarian Ovarian Cancer Cases and Controls)	Bavaria, Germany	Hospital based	204	56.0 (0.7)	229	58.0 (0.7)
HAW (Hawaiian Ovarian Cancer Study)	Hawaii, USA	Population-based case-control	64	55.0 (1.3)	152	56.8 (0.9)
MAL (The Danish Malignant Ovarian Tumor Study)	Denmark		348	59.9 (0.6)	893	56.8 (0.4)
NCO (North Carolina Ovarian Cancer Study)	North Carolina, USA	Population-based case-control	520	57.8 (0.5)	582	55.2 (0.4)
POC (Polish Ovarian Cancer Study)	Szczecin, Poznan, Opole and Rzeszow, Poland	Population-based case-control	545	55.0 (0.5)	525	57.5 (0.5)
SEA (UK SEARCH Ovarian Cancer Study)	United Kingdom		936	56.0 (0.3)	1198	55.0 (0.3)
STA (Genetic Epidemiology of Ovarian Cancer)	California, USA	Population-based case-control	265	51.4 (0.7)	338	48.2 (0.6)
UKO (UK Ovarian Cancer Population Study)	United Kingdom		634	61.0 (0.4)	998	64.9 (0.3)
USC (Los Angeles County Case-Control Studies of Ovarian Cancer)	California, USA	Population-based case-control	379	58.0 (0.5)	519	56.3 (0.5)
POOLED			4946	57.3 (0.2)	6582	57.2 (0.1)

**Figure 1:**
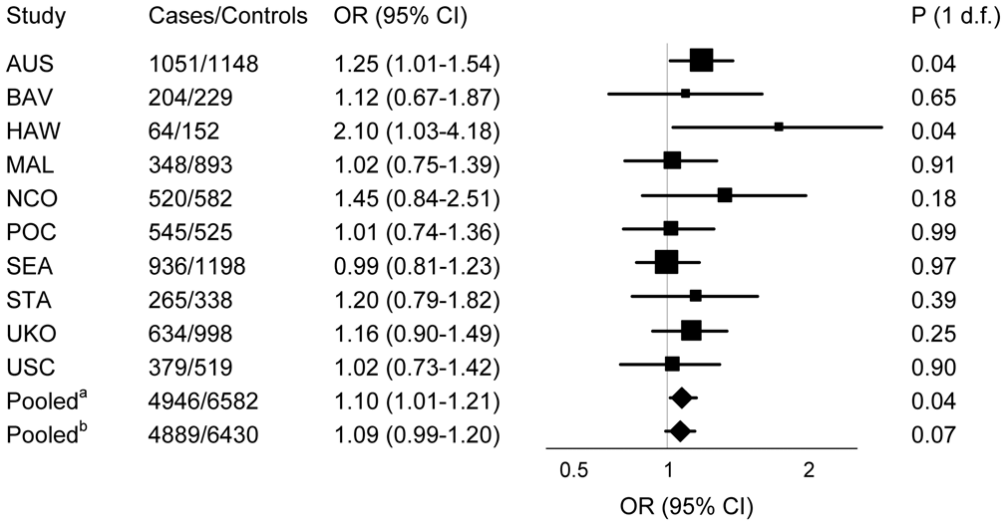
Association of the *ESR2* rs1271572 with invasive ovarian carcinoma risk. Forest plot of the ORs and 95% CIs for invasive ovarian carcinoma risk associated with carriage of the *ESR2* rs1271572 *TT* genotype versus *GG/GT* genotypes (recessive genetic model). P for heterogeneity of the association of the *ESR2* rs1271572 with risk by study = 0.60. Pooled^a^ OR for all studies combined was 1.10 (95% CI: 1.01–1.21; p = 0.04). Pooled^b^ OR for all studies excluding HAW was 1.09 (95% CI: 0.99–1.20; p = 0.07).

In all studies combined, women with the *TT* genotype had increased ovarian carcinoma risk [odds ratio (OR) = 1.10; 95% confidence interval (CI): 1.01–1.21; p = 0.04] compared to carriers of any *G* allele (recessive genetic model) (Figure 1 and Table S2). This OR was reduced to 1.09 (CI: 0.99–1.20; p = 0.07) after excluding the Hawaii data. In addition to the HAW study, the association was also statistically significant among AUS study participants (OR = 1.25; 95% CI: 1.01–1.54; p = 0.04) (Figure 1 and Table S2). Excluding the AUS study, where the genotype deviated from Hardy-Weinberg equilibrium (HWE), slightly attenuated the association of the rs1271572 SNP with ovarian carcinoma risk (OR = 1.07; 95% CI: 0.97–1.19; p = 0.09).

**Figure 2:**
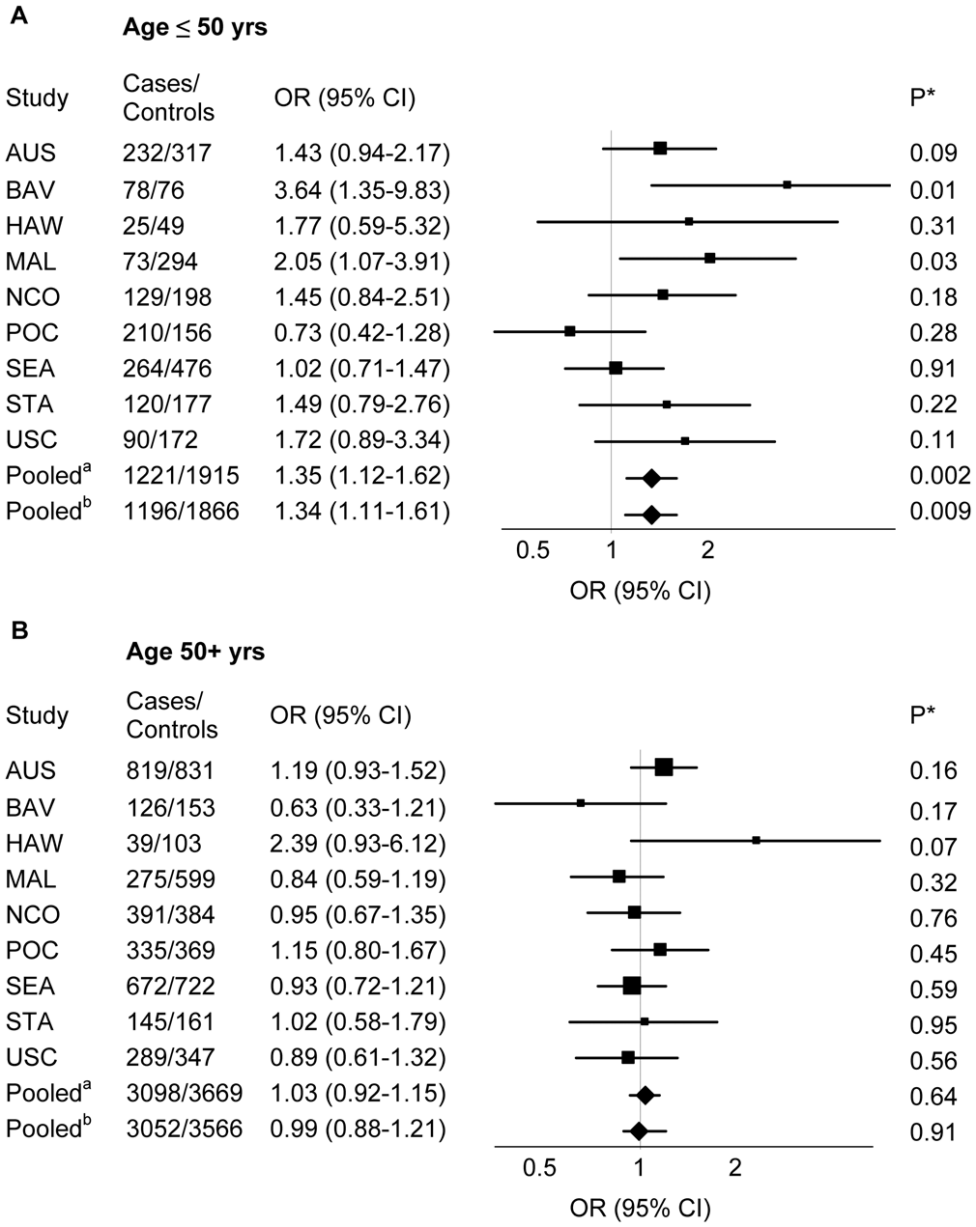
Association of the *ESR2* rs1271572 with invasive ovarian carcinoma risk in subgroups by age. Forest plot of the ORs and 95% CIs for invasive ovarian carcinoma risk associated with carriage of the *ESR2* rs1271572 *TT* genotype versus *GG/GT* genotypes (recessive genetic model) in subgroups of women ≤50 (A) versus > 50 years (B) of age. Pooled^a^ OR for all studies combined among women ≤50 years was 1.35 (95% CI: 1.12–1.62; p = 0.002); p for heterogeneity among studies = 0.19. Pooled^b^ OR for all studies excluding HAW was 1.34 (95% CI: 1.11–1.61; p = 0.009); p for heterogeneity among studies = 0.29. P for interaction between *ESR2* rs1271572 and age = 0.02.

There was a significant interaction between genotype and age (p = 0.02) (Figure 2 and Table S3). Among younger women (≤50 years old), women with the *TT* genotype had a 35% increased risk of ovarian carcinoma (95% CI: 1.2–1.67; p = 0.002) compared to *G* allele carriers. No genetic associations were observed among women >50 years old (OR = 1.01; CI: 0.89–1.14; p = 0.91). The association of the *TT* genotype with risk among women ≤50 years old was also statistically significant in two individual studies, BAV (OR = 3.64; CI: 1.35–9.83; p = 0.01) and MAL (OR = 2.05; CI: 1.07–3.91; p = 0.03) and in the pooled analysis when HAW data were excluded (OR = 1.34; 95% CI: 1.11–1.61; p = 0.009). The association of the *TT* genotype with risk was also higher in premenopausal (OR = 1.20; CI: 0.99–1.45; p = 0.06) compared to postmenopausal women (OR = 1.10; CI: 0.98–1.24; p = 0.12), although the test for heterogeneity in effect was not significant (p = 0.82). No heterogeneity of the associations was observed among studies for any of the models (p range: 0.13–0.84). No effect modification by gravidity, parity, menopausal status, and use of contraceptive and menopausal hormones was found for the association of rs1271572 with risk (data not shown). No heterogeneity of effects was observed by tumor histology (p range: 0.20–0.87) (Table S4) or stage at diagnosis (p = 0.87) (data not shown).

## Discussion

In this pooled analysis of ten case-control studies, we found a modest association of the rs1271572 *TT* genotype with ovarian carcinoma risk, particularly among younger women ≤50 years of age. This significant association of rs1271572 with the risk of ovarian cancer among younger women might result from the higher concentrations of circulating estrogens among pre- and perimenopausal women. By analogy, we hypothesize that among postmenopausal women with lower estrogen levels, *ESR2* functional variation would not be an important contributor to risk.

A potential causal association of rs1271572 with the risk of ovarian cancer is supported by the finding that it maps to the promoter of the *ESR2* gene, near (−53 bp upstream) the AP-4/MyoD binding site [14], a region of predicted intense transcription factor binding that might influence gene expression [7]. Transcription factor AP-4 contains multiple dimerization domains that function to promote ERβ/ERβ homodimer formation [15]. The MyoD transcription regulator promotes the resistance of ER to proteolytic degradation [16]. Therefore, the rs1271572 sequence variation might reduce the anti-proliferative effects of ERβ proposed by altering *ESR2* responsiveness to transcription regulators.

Although the specific role of ERβ in carcinogenesis is not known, there is convincing evidence that ERβ inhibits proliferation and motility of ovarian cancer cells and plays an important role in apoptosis [8], [17]. In a study by Lindgren et al.[9], overexpressing ERβ in an ovarian adenocarcinoma cancer cell line PEO14 led to a 50% reduction in proliferative capacity. An antitumor role of ERβ in SK-OV-3 ovarian cancer cells that do not express functional ERα has been reported by Treeck et al. [18]. Down-regulation of ERβ has also been noted in breast, colon, and prostate cancers [19]–[21], malignancies that share some etiologic features with ovarian cancer [22].

Previously, Pierce et al. evaluated [23] *ESR2* variation in relation to ovarian cancer risk, using a haplotype approach. No statistically significant associations were found, although one haplotype was associated with an increased risk of invasive clear cell carcinoma. While the rs1271572 SNP was not genotyped in this study, it was in the haplotype block represented by the rs1271530 SNP which is in a strong linkage disequilibrium (r^2^ = 0.9) with rs1271572 based on the HapMAP data among whites. A previous genome-wide association study (GWAS) [24] of ovarian cancer susceptibility, had limited power to detect modest genetic associations: phase I included 1817 cases and 2353 and had a 57% power to detect ORs as low as 1.10 under a log-additive model and 23% under a recessive model. Sun et al. [25] found an association of the rs1271572 polymorphism with the risk of prostate cancer among Chinese men.

The strengths of this investigation are the population-based nature of the studies included and the stringent genotyping quality control procedures established by the OCAC. A further strength is the large sample size and the relatively high allele frequency. The False Positive Report Probability [26] for our sample size and power was noteworthy (<0.5 level) for an OR of 1.10 for moderate to high prior probabilities (≥0.10). Population stratification might have influenced the results of our investigation. To minimize the population stratification effects, this study included only white non-Hispanic women from developed countries with comparable ovarian cancer incidence rates. Nonetheless, false positive findings are possible and further replication studies are being conducted to confirm the association. Furthermore, our statistical power was limited to study gene-environment interactions.

In conclusion, the results of this pooled analysis suggest that the rs1271572 SNP in the *ESR2* gene may influence the risk of invasive ovarian carcinoma, especially among younger women.

## Materials and Methods

### Ethics Statement

All studies were approved by the review boards and ethics committees of their parent institutions, and written informed consent was obtained from all participants. In addition, Duke University has Institutional Review Board approval as a data coordinating center. All data were analyzed anonymously.

### Study Design and Population

This pooled analysis of nine population-based studies from Australia [the Australian Ovarian Cancer study and the Australian Cancer Study: Ovarian Cancer (AUS)], the United States [Genetic Epidemiology of Ovarian Cancer Study, Stanford University, California (STA); Hawaiian Ovarian Cancer Study, Honolulu, Hawaii (HAW); the North Carolina Ovarian Cancer Study, North Carolina (NCO); the University of Southern California Case-Control Studies of Ovarian Cancer, Los Angeles County, California (USC)], and Europe [MALOVA Ovarian Cancer Case-Control Study, Danish Cancer Society, Denmark (MAL); Studies of Epidemiology and Risk Factors in Cancer Heredity, United Kingdom (SEA); the United Kingdom Ovarian Cancer Population Study (UKO); the Polish Ovarian Cancer study, Poland (POC)] and one hospital-based study [Bavaria Case-Control Study (BAV)] included 4946 cases with primary histologically-confirmed invasive ovarian carcinoma and 6582 controls. Control subjects were randomly selected from the same geographical areas as cases. Eligibility criteria for controls included age 18 years or older, no prior history of ovarian cancer, and having at least one intact ovary. All cases and controls were non-Hispanic white women. A detailed description of the studies has been previously published and is summarized in Table 1 and Table S5. Epidemiological data were collected using structured questionnaires that included socio-demographic and health-related information, menstrual, reproductive, and gynecological histories. OCAC members submitted their epidemiological data to Duke University where the variables have been reviewed, cleaned, and merged. Histology and stage data were available for 91% and 93% of cases, respectively. Information on menopausal status was available for 91% of women. Data on gravidity and parity was available for 86% (n = 9878 and n = 9950, respectively) of women (it was missing for all POC study participants and 5% of women from other studies combined). Data on use of contraceptive hormones were available for 83% (n = 9580) of women (the data were missing for POC and BAV women and for 5% of women from all other studies). Data on use of menopausal hormones were available only for 30% (n = 1549) of postmenopausal women.

### Genotyping

Genotyping for the AUS study was performed using the Sequenom iPlex gold genotyping platform (Sequenom, Inc.). All other studies used TaqMan allelic discrimination assay (TaqMan; Applied Biosystems). We used the following quality control criteria that were established by the OCAC to measure the acceptability of the genotyping results: (1) >3% sample duplicates included, (2) concordance rate for duplicate samples ≥98%, (3) overall call rate (by study) >95% and (4) call rate >90% for each 384-well plate (5) no more than 5% difference in call rates between cases and controls, and (6) cases and controls intermixed on each plate. In addition, consistency across laboratories was confirmed by genotyping a common set of 95 DNAs (90 CEPH trios and five duplicate samples; HAPMAPPT01 provided by Coriell) with the requirement of >98% concordance in genotype calls. All ten studies met each of the criteria. Genotyping quality was also assessed using tests for Hardy-Weinberg equilibrium (HWE). The genotype distribution for the SNP among controls was consistent with HWE in all but one study (AUS; p = 0.03). Exclusion of this study did not appreciably affect the reported results.

Gene and allele nomenclature was according to the National Center of Biotechnology Information.

### Statistical analysis

Statistical analyses were performed using the SAS statistical package (SAS release 9.2, SAS Institute Inc., Cary, NC). The chi-square test for association was used to compare the allele frequency distributions among controls across studies, and the chi-square test for goodness-of-fit was used to test consistency with the HWE for each study and overall. The association of the rs1271572 polymorphism with ovarian carcinoma risk was assessed using multivariate logistic regression models. ORs and 95% CIs were estimated separately for heterozygous and homozygous variant *T* allele carriers, using women with the *GG* genotype as the reference group. We also performed genetic analyses testing a log-additive model in which genotype was categorized by three levels (0, 1 and 2) representing number of variant alleles. In addition, we compared risk among women with the *TT/GT* versus *GG* genotypes (testing a dominant genetic model) and among women with the *TT* genotype versus the *GG/GT* genotypes (testing a recessive genetic model). Based on the Akaike Information Criterion (AIC), the recessive model provided the best fit for the data. All models are presented in tables for comprehensiveness (supporting material).

Analyses were conducted for each study separately and for all studies combined. All models were adjusted for age to control for a potential residual confounding by imperfect matching. Heterogeneity of effects by study was examined using two different methods. First, we included study site as a fixed effect covariate and evaluated heterogeneity of the association of the rs1271572 SNP with risk by study, using a Wald test of the genotype-study interaction term. Second, we included study site as a random effect using SAS GLIMMIX procedure. No heterogeneity was observed in any of the models, and the results were the same. We also conducted the analysis excluding data from the Hawaii study which initially nominated the SNP for further validation. The association of the rs1271572 with ovarian carcinoma risk by histologic type (serous, mucinous, endometrioid, clear cell, mixed, other specified epithelial, undifferentiated and other unknown epithelial) was studied using polytomous logistic regression; the estimated ORs among histological types were compared using the Wald test. Heterogeneity of the association of the rs1271572 genotype with risk by age, gravidity, parity, menopausal status, and use of contraceptive and menopausal hormones was evaluated using the Wald test comparing group-specific parameters for the rs1271572 genotype in the logistic regression models. All p-values were based on two-tailed tests. We evaluated statistical significance at the 5% level.

## Supporting Information

Table S1 *ESR2* rs1271572 genotype and minor allele frequencies (MAF) among non-Hispanic white women by study.
(DOC)

Table S2 *ESR2* rs1271572 genotype associations with ovarian cancer risk by study.
(DOC)

Table S3 *ESR2* rs1271572 genotype associations with ovarian cancer risk by study stratified by age (≤50 versus >50 years).
(DOC)

Table S4 Association of the *ESR2* rs1271572 genotype with ovarian carcinoma risk by histological type.
(DOC)

Table S5 Case ascertainment and selection of controls.
(DOC)
