# Supplementary material for: Estrogen Receptor Beta rs1271572 Polymorphism and Invasive Ovarian Carcinoma Risk: Pooled Analysis within the Ovarian Cancer Association Consortium
Source: PLoS One. 2011 Jun 6;6(6):e20703. doi: 10.1371/journal.pone.0020703 (PMC3108970; doi:10.1371/journal.pone.0020703)
Supplement: Table S1 — ESR2 rs1271572 genotype and minor allele frequencies (MAF) among non-Hispanic white women by study. (DOC) [file pone.0020703.s001.doc]

**Table S1.** *ESR2* rs1271572 genotype and minor allele frequencies (MAF) among non-Hispanic white women by study

| Study | Cases (N) | Controls (N) | *ESR2* rs1271572 genotype | | | | | | MAF among controls | *P** |
| --- | --- | --- | --- | --- | --- | --- | --- | --- | --- | --- |
|  |  |  | N (%) Cases | | | N (%) Controls | | |  |  |
|  |  |  | *GG* | *GT* | *TT* | *GG* | *GT* | *TT* |  |  |
| AUS | 1051 | 1148 | 312 (30) | 514 (49) | 225 (21) | 339 (30) | 603 (52) | 206 (18) | 0.44 | *0.03* |
| BAV | 204 | 229 | 62 (30) | 107 (53) | 35 (17) | 75 (33) | 118 (51) | 36 (16) | 0.41 | *0.35* |
| HAW | 64 | 152 | 18 (28) | 28 (44) | 18 (28) | 43 (28) | 86 (57) | 23 (15) | 0.43 | *0.06* |
| MAL | 348 | 893 | 92 (27) | 185 (53) | 71 (20) | 257 (29) | 463 (52) | 173 (19) | 0.45 | *0.17* |
| NCO | 520 | 582 | 150 (29) | 262 (50) | 108 (21) | 181 (31) | 283 (49) | 118 (20) | 0.45 | *0.70* |
| POC | 545 | 525 | 177 (32) | 266 (49) | 102 (19) | 176 (33) | 250 (48) | 99 (19) | 0.43 | *0.54* |
| SEA | 936 | 1198 | 284 (30) | 461 (49) | 191 (20) | 353 (29) | 599 (50) | 246 (21) | 0.46 | *0.78* |
| STA | 265 | 338 | 80 (30) | 130 (49) | 55 (21) | 105 (31) | 175 (52) | 58 (17) | 0.43 | *0.30* |
| UKO | 634 | 998 | 178 (28) | 318 (50) | 138 (22) | 294 (29) | 507 (51) | 197 (20) | 0.45 | *0.42* |
| USC | 379 | 519 | 133 (35) | 168 (44) | 78 (21) | 147 (28) | 268 (52) | 104 (20) | 0.46 | *0.36* |
| P† |  |  |  |  | *0.62* |  |  | *0.69* |  |  |

*P for the chi-square test assessing deviation of genotype frequencies among controls from those expected under Hardy-Weinberg equilibrium.

†P for heterogeneity of genotype distribution by study was assessed using likelihood ration chi-square test.
