# Supplementary material for: Estrogen Receptor Beta rs1271572 Polymorphism and Invasive Ovarian Carcinoma Risk: Pooled Analysis within the Ovarian Cancer Association Consortium
Source: PLoS One. 2011 Jun 6;6(6):e20703. doi: 10.1371/journal.pone.0020703 (PMC3108970; doi:10.1371/journal.pone.0020703)
Supplement: Table S2 — ESR2 rs1271572 genotype associations with ovarian cancer risk by study. (DOC) [file pone.0020703.s002.doc]

**Table S2.** *ESR2* rs1271572 genotype associations with ovarian cancer risk by study

| Study | Heterozygotes and rare allele homozygotes* | | | Log-additive model | | Recessive model* | |
| --- | --- | --- | --- | --- | --- | --- | --- |
|  | *GT* | *TT* | P (2 d.f.) | Per allele OR | P for trend | *TT* vs.*GG+GT* | P |
|  | OR (95% CI)† | OR (95% CI)† |  | (95% CI)† |  | OR (95% CI)† |  |
| AUS | 0.93 (0.76-1.13) | 1.19 (0.93-1.52) | *0.09* | 1.07 (0.95-1.21) | *0.26* | 1.25 (1.01-1.54) | *0.04* |
| BAV | 1.10 (0.72-1.69) | 1.20 (0.67-2.13) | *0.82* | 1.10 (0.83-1.45) | *0.53* | 1.12 (0.67-1.87) | *0.65* |
| HAW | 0.78 (0.39-1.57) | 1.80 (0.78-4.14) | *0.10* | 1.30 (0.84-2.01) | *0.24* | 2.10 (1.03-4.18) | *0.04* |
| MAL | 1.09 (0.81-1.46) | 1.08 (0.74-1.55) | *0.85* | 1.04 (0.87-1.25) | *0.67* | 1.02 (0.75-1.39) | *0.91* |
| NCO | 1.13 (0.81-1.60) | 1.13 (0.81-1.60) | *0.67* | 1.07 (0.90-1.27) | *0.43* | 1.45 (0.84-2.51) | *0.18* |
| POC | 1.04 (0.79-1.37) | 1.03 (0.72-1.45) | *0.96* | 1.02 (0.86-1.21) | *0.85* | 1.01 (0.74-1.36) | *0.99* |
| SEA | 0.95 (0.78-1.16) | 0.97 (0.76-1.24) | *0.90* | 0.98 (0.87-1.11) | *0.76* | 0.99 (0.81-1.23) | *0.97* |
| STA | 1.04 (0.71-1.51) | 1.23 (0.76-1.98) | *0.67* | 1.10 (0.87-1.39) | *0.42* | 1.20 (0.79-1.82) | *0.39* |
| UKO | 1.07 (0.84-1.36) | 1.21 (0.90-1.63) | *0.44* | 1.10 (0.95-1.27) | *0.21* | 1.16 (0.90-1.49) | *0.25* |
| USC | 0.70 (0.52-0.95) | 0.83 (0.57-1.20) | *0.07* | 0.88 (0.73-1.07) | *0.19* | 1.02 (0.73-1.42) | *0.90* |
| POOLED | 0.98 (0.90-1.06) | 1.09 (0.98-1.21) | *0.10* | 1.04 (0.98-1.09) | *0.20* | 1.10 (1.01-1.21) | *0.04* |
| Excluding HAW | 0.98 (0.90-1.07) | 1.08 (0.97-1.20) | *0.18* | 1.03 (0.98-1.09) | *0.26* | 1.09 (0.99-1.20) | *0.07* |
| *P*‡ |  |  | *0.63* |  | *0.74* |  | *0.60* |

* *GG* genotype was used as the reference category.

† Odds ratios (OR) and 95% confidence intervals (CI) from the unconditional logistic regression models adjusted for age and, in combined analyses, by study.

‡ P for heterogeneity of the association of the rs1271572 SNP with risk by study was estimated using a Wald test of the genotype-study interaction terms.
