# Supplementary material for: Estrogen Receptor Beta rs1271572 Polymorphism and Invasive Ovarian Carcinoma Risk: Pooled Analysis within the Ovarian Cancer Association Consortium
Source: PLoS One. 2011 Jun 6;6(6):e20703. doi: 10.1371/journal.pone.0020703 (PMC3108970; doi:10.1371/journal.pone.0020703)
Supplement: Table S3 — ESR2 rs1271572 genotype associations with ovarian cancer risk by study stratified by age (≤50 versus >50 years). (DOC) [file pone.0020703.s003.doc]

**Table S3.** *ESR2* rs1271572 genotype associations with ovarian cancer risk by study stratified by age (≤ 50 versus >50 years)

| Study | Cases/controls | Heterozygotes and rare allele homozygotes* | | | Log-additive model | | Recessive model* | |
| --- | --- | --- | --- | --- | --- | --- | --- | --- |
|  |  | GT | TT | P (2 d.f.) | Per allele | P for trend | TT vs. GG+GT | P |
|  |  | OR (95% CI)† | OR (95% CI)† |  | OR (95% CI)† |  | OR (95% CI)† |  |
| Women ≤ 50 years old | | | | | | | | |
| AUS | 232/317 | 0.89 (0.60-1.33) | 1.32 (0.81-2.17) | 0.21 | 1.00 (0.81-1.74) | 0.99 | 1.43 (0.94-2.17) | 0.09 |
| BAV | 78/76 | 0.62 (0.30-1.26) | 2.76 (0.94-8.08) | 0.02 | 1.31 (0.82-2.08) | 0.26 | 3.64 (1.35-9.83) | 0.01 |
| HAW | 25/49 | 0.61 (0.19-1.94) | 1.31 (0.36-4.77) | 0.42 | 1.13 (0.58-2.20) | 0.72 | 1.77 (0.58-5.32) | 0.31 |
| MAL | 73/294 | 1.05 (0.57-1.91) | 2.11 (1.00-4.45) | 0.09 | 1.40 (0.96-2.06) | 0.08 | 2.05 (1.07-3.91) | 0.03 |
| NCO | 129/198 | 1.46 (0.85-2.50) | 1.84 (0.97-3.51) | 0.16 | 1.36 (0.99-1.88) | 0.06 | 1.45 (0.84-2.51) | 0.18 |
| POC | 210/156 | 1.18 (0.74-1.88) | 0.81 (0.43-1.52) | 0.44 | 0.94 (0.70-1.28) | 0.72 | 0.73 (0.42-1.28) | 0.28 |
| SEA | 264/476 | 1.34 (0.93-1.93) | 1.24 (0.79-1.95) | 0.12 | 1.13 (0.90-1.41) | 0.29 | 1.02 (0.71-1.47) | 0.91 |
| STA | 120/177 | 0.79 (0.45-1.39) | 1.26 (0.61-2.64) | 0.33 | 1.08 (0.75-1.56) | 0.68 | 1.49 (0.79-2.79) | 0.22 |
| USC | 90/172 | 0.68 (0.38-1.21) | 1.36 (0.64-2.67) | 0.19 | 1.08 (0.74-1.57) | 0.70 | 1.72 (0.89-3.34) | 0.11 |
| POOLED | 1221/1915 | 1.03 (0.87-1.22) | 1.37 (1.11-1.70) | 0.006 | 1.16 (1.04-1.29) | 0.008 | 1.35 (1.12-1.62) | 0.002 |
| Excluding HAW | 1196/1866 | 1.04 (0.88-1.24) | 1.37 (1.10-1.70) | 0.01 | 1.16 (1.04-1.29) | 0.009 | 1.34 (1.11-1.61) | 0.009 |
| P‡ |  |  |  | 0.13 |  | 0.84 |  | 0.19 |
| Women > 50 years old | | | | | | | | |
| AUS | 819/831 | 0.94 (0.76-1.18) | 1.15 (0.87-1.52) | 0.33 | 1.06 (0.92-1.21) | 0.44 | 1.19 (0.93-1.52) | 0.16 |
| BAV | 126/153 | 1.51 (0.88-2.60) | 0.83 (0.39-1.73) | 0.13 | 0.99 (0.69-1.40) | 0.94 | 0.63 (0.33-1.21) | 0.17 |
| HAW | 39/103 | 0.90 (0.37-2.19) | 2.23 (0.74-6.75) | 0.19 | 1.44 (0.81-2.56) | 0.22 | 2.39 (0.93-6.12) | 0.07 |
| MAL | 275/599 | 1.09 (0.77-1.53) | 0.89 (0.58-1.35) | 0.55 | 0.95 (0.77-1.17) | 0.62 | 0.84 (0.59-1.19) | 0.32 |
| NCO | 391/384 | 0.99 (0.72-1.38) | 0.94 (0.63-1.42) | 0.95 | 0.97 (0.80-1.19) | 0.79 | 0.95 (0.67-1.35) | 0.76 |
| POC | 335/369 | 0.97 (0.69-1.36) | 1.14 (0.75-1.72) | 0.74 | 1.05 (0.86-1.30) | 0.62 | 1.15 (0.80-1.67) | 0.45 |
| SEA | 672/722 | 0.81 (0.64-1.03) | 0.82 (0.60-1.10) | 0.21 | 0.89 (0.77-1.04) | 0.14 | 0.93 (0.72-1.21) | 0.59 |
| STA | 145/161 | 1.24 (0.74-2.06) | 1.15 (0.61-2.16) | 0.72 | 1.09 (0.80-1.49) | 0.59 | 1.02 (0.58-1.79) | 0.95 |
| USC | 289/347 | 0.70 (0.48-0.99) | 0.72 (0.47-1.12) | 0.12 | 0.83 (0.67-1.04) | 0.10 | 0.90 (0.61-1.32) | 0.59 |
| POOLED | 3091/3669 | 0.94 (0.84-1.05) | 0.97 (0.85-1.12) | 0.59 | 0.99 (0.94-1.06) | 0.97 | 1.01 (0.89-1.14) | 0.91 |
| Excluding HAW | 3052/3566 | 0.94 (0.84-1.06) | 0.96 (0.83-1.10) | 0.60 | 0.98 (0.91-1.05) | 0.48 | 0.99 (0.88-1.12) | 0.91 |
| P‡ |  |  |  | 0.30 |  | 0.43 |  | 0.60 |
| P§ |  |  | 0.04 |  | 0.02 |  | 0.02 |  |

* *GG* genotype was used as the reference category.

† Odds ratios (OR) and 95% confidence intervals (CI) from the unconditional logistic regression models adjusted for age and, in combined analyses, by

study.

‡ P for heterogeneity of the association of the rs1271572 SNP with risk by study was estimated using a Wald test of the genotype-study interaction term.

*§* P for interaction of the association of the rs1271572 SNP with risk by age (≤ 50 versus >50 years old) was estimated using a Wald test of the genotype-age group interaction term.

Note: All UKO cases were older than 50 years old.
