# Supplementary material for: Estrogen Receptor Beta rs1271572 Polymorphism and Invasive Ovarian Carcinoma Risk: Pooled Analysis within the Ovarian Cancer Association Consortium
Source: PLoS One. 2011 Jun 6;6(6):e20703. doi: 10.1371/journal.pone.0020703 (PMC3108970; doi:10.1371/journal.pone.0020703)
Supplement: Table S4 — Association of the ESR2 rs1271572 genotype with ovarian carcinoma risk by histological type. (DOC) [file pone.0020703.s004.doc]

**Table S4.** Association of the *ESR2* rs1271572 genotype with ovarian carcinoma risk by histological type

| Histological type | All | *GG* | *GT* | *TT* | *GT* | | *TT* | | *P trend* | *TT vs. any G* | |
| --- | --- | --- | --- | --- | --- | --- | --- | --- | --- | --- | --- |
|  | N | | | | OR (95% CI)* | P* | OR (95% CI)* | P* |  | OR (95%CI)* | P* |
| Serous | 2635 | 800 (30) | 1309 (50) | 526 (20) | 0.97 (0.87-1.08) | *0.55* | 1.04 (0.91-1.19) | *0.54* | *0.66* | 1.06 (0.95-1.19) | *0.30* |
| Mucinous | 405 | 121 (30) | 201 (50) | 83 (20) | 0.99 (0.77-1.30) | *0.93* | 1.09 (0.81-1.46) | *0.57* | *0.62* | 1.10 (0.85-1.46) | *0.48* |
| Enodmetrioid | 706 | 213 (30) | 349 (49) | 144 (20) | 0.97 (0.81-1.16) | *0.74* | 1.05 (0.84-1.32) | *0.66* | *0.48* | 1.06 (0.88-1.29) | *0.54* |
| Clear cell | 368 | 113 (31) | 179 (48) | 76 (21) | 0.94 (0.73-1.19) | *0.59* | 1.04 (0.77-1.41) | *0.79* | *0.88* | 1.09 (0.84-1.41) | *0.53* |
| Mixed epithelial | 137 | 44 (32) | 65 (48) | 28 (20) | 0.83 (0.56-1.23) | *0.35* | 0.99 (0.61-1.62) | *0.99* | *0.85* | 1.12 (0.73-1.71) | *0.61* |
| Other specified epithelial | 205 | 59 (29) | 97 (47) | 49 (24) | 1.04 (0.74-1.45) | *0.84* | 1.35 (0.91-2.01) | *0.13* | *0.16* | 1.32 (0.95-1.85) | *0.10* |
| Other unknown epithelial | 490 | 136 (28) | 239 (49) | 115 (23) | 1.06 (0.85-1.33) | *0.59* | 1.33 (1.03-1.73) | *0.03* | *0.03* | 1.28 (1.01-1.31) | *0.03* |
| P† |  |  |  |  |  |  | *0.87* |  | *0.20* | *0.33* |  |

Note: GG genotype was used as a reference group.

* Odds ratio (OR), 95% confidence interval (CI), and pair-wise p-values from unconditional logistic regression model, adjusted for age (continuous) and study.

†P values for heterogeneity of the main effects among histological types were calculated using pair-wise Wald test for case groups.
