# Supplementary material for: Estrogen Receptor Beta rs1271572 Polymorphism and Invasive Ovarian Carcinoma Risk: Pooled Analysis within the Ovarian Cancer Association Consortium
Source: PLoS One. 2011 Jun 6;6(6):e20703. doi: 10.1371/journal.pone.0020703 (PMC3108970; doi:10.1371/journal.pone.0020703)
Supplement: Table S5 — Case ascertainment and selection of controls. (DOC) [file pone.0020703.s005.doc]

**Table S5.** Case ascertainment and selection of controls

| Study Name | Case ascertainment | Selection of controls |
| --- | --- | --- |
|
|
| AUS | Diagnosed from 2002 onwards; recruited through surgical treatment centres throughout Australia and cancer registries of Queensland, Southern and Western Australia, New South Wales, and Victoria | Randomly selected from Commonwealth Electoral roll |
| BAV | Hospital based study from Erlangen, Northern Bavaria, Germany. Recruitment from May 2002 to August 2008 | Random selected women from Erlangen, Northern Bavaria, Germany. Recruitment from May 2002 to August 2008 |
| HAW | Rapid case ascertainment through Hawaii Tumor Registry from 1993 onwards | Randomly selected from Hawaii Department of Health Annual Survey of representative households; matched to cases for age (5-year categories) and ethnicity |
| MAL | Cases (35-79 years) diagnosed 1994 -1999 from municipalities of Copenhagen and Frederiksberg and surrounding counties | Population based: random sample of female population selected from the same areas as cases |
| NCO | Cases from 1999 onwards identified from 48 counties within the region by rapid-case ascertainment. | Controls identified from same counties as cases and frequency matched to cases for age and race |
| POC | Cases diagnosed between 1998-2006 from Szczecin, Poznan, Opole, and Rzeszow, Poland | Frequency matched to cases on age and study site and randomly selected within matching strata from the same population |
| SEA | Cases < 70 years from East Anglian, West Midlands and Trent regions of England. Prevalent cases diagnosed 1991-1998; incident cases from 1998 onwards | Selected from the EPIC-Norfolk cohort of 25,000 individuals aged 45-74 based in the same geographical region as cases |
| STA | Consecutive cases diagnosed from 1997-2002 in Greater Bay Area Cancer Registry San Francisco. | Population- and family-based |
| UKO | Cases attending ten major Gynaecological Oncology centers in England, Wales and Northern Ireland from 2006 onwards | Postmenopausal women from the general population participating in the UK Collaborative Trial of Ovarian cancer Screening |
| USC | Rapid case ascertainment through Los Angeles Cancer Surveillance program from 1993 onwards | Neighborhood recruits, frequency matched for age and ethnicity |
